# Medical Education

**Published:** 1882-06

**Authors:** 


### Editorial.

### Medical Education.

In the two preceding numbers of the Journal are contained the first two of a series of editorials of which this is the third and concluding. In the former, we referred to the comparative low standard of medical education, and the causes which we recognize as having been instrumental in bringing this about. We now proceed earnestly to call attention to the only remedy which in our opinion can be efficient.

The standard of medical education must be decidedly elevated. Necessarily this will result in a marked diminution of the number of candidates and still a greater lessening of the number of graduates. Such has been the result in certain European countries where the experiment has been made. The latter by diminishing the excessive rivalry and competition for livelihood in so much affords the time as the former will foster the inclination for scientific study and work. That the former object is desirable is clearly shown by the following extract from the *Medicai Record*, entitled, "What is he going to do?"

"The graduation of some five or six thousand young medical men during the present spring is an event of deep importance to the profession. What are they going to do? is the question asked, not only by the parties especially interested, but by the profession and public at large. The New York *Times*, which always discusses medical questions with more than ordinary fairness and intelligence, has recently applied itself to the problem just suggested. There is now, it says, in the United States one physician to every five hundred inhabitants. The ratio of sick to well during the year is estimated at about twenty per one thousand, including the paupers. This would give about ten patients to each practitioner; but of these many are too poor to pay, and many do not call in a physician, so that five or six patients are all that can be allowed for each medical man.

Another method studying the question is as follows: The ratio of deaths is, for the whole country, not far from twenty per one thousand annually. For every death there are about twenty-five cases of sickness, or five hundred cases per one thousand annually. This would give an average of two hundred and fifty patients per year for each physician. But this number must be reduced nearly one-half, by subtracting those who do not pay, and those who are not ill enough to need a physician. With the present number of physicians, therefore, there would be two or three patients per week for each, if they were equally divided.

What need is there, then, for six thousand new doctors annually? If they do what they expect, if they practice and prosper, it must be at the expense of those already in the field."

In the present system we find that practically the control is vested in the professors of the various colleges. Their decision is practically final. If an overbold curator impertinently offers to question this, he is summarily dropped. Curators are in fact figure heads, well masked to deceive the public. The reputation and, even more, the financial success of medical colleges, as at present constituted, are unhappily directly dependent upon the number of their graduates. The temptation to yield the higher interests of the profession and the public to the more personal consideration of pecuniary success, is but rarely withstood. The professors are in fact, and with an occasional exception, deservedly the leaders of the profession. They are, as a class, fully as honorable as their brethren in the profession and no more subject to the greed of money getting, or to the acquirement of reputation by questionable resorts. And yet in fact we find the temptation too great, apparently irresistible. To remove this temptation should be the unanimous endeavor, not alone of the profession, but at least equally of the general public, who are even more vitally interested in the matter of more competent medical advisers. The control must be taken out of the hands of those pecuniarily or otherwise selfishly interested, and placed with those who, looking only to the public good, shall decide without prejudice or favor. State or national boards, appointed in the manner and wielding power as uninfluenced as that of our judges of supreme courts, is the obvious ultimatum. Sooner or later public opinion will force this reformation. And it behooves the profession to see to it, that they remain in the van, and, not being disgracefully inactive, have it forced upon them.

The former incentive of degenerative tendency being removed, the rivalry of colleges will assume a higher and more commendable character. An impartial board of final decision existing, the various colleges within its jurisdiction and sending applicants for approval, will soon discover the new sources of reputation to be the advantages offered for study, and the character of the students recommended for the favorable consideration of the board of final resort. As is now the case with the army and naval boards, the small percentage of rejections will then constitute the honorable record of merit. The incentive will then be to increase the advantages for the study of disease, and to strive at more thorough and availing instruction. The various colleges will then find their true level, and in due time the profession will show the result in the speedy disappearance of quackery and the increased zeal for honest scientific work.

That the profession itself is mainly, if not solely, responsible for the present unsatisfactory state of medical education, and that it having at present, as it always has had, the power to remedy, either ignores or still worse, fosters this disgrace, almost passes belief. Unendurable would seem the oppression, were the origin of this evil and the power of remedy in other hands. This matter may no longer, and we have reason to believe will no longer be delayed. When the ball is put in motion, we call upon all to help it along.

